# Tribological Properties and Lubrication Mechanisms of Water-Based Nanolubricants Containing TiO_2_ Nanoparticles during Micro Rolling of Titanium Foils

**DOI:** 10.3390/ma17010170

**Published:** 2023-12-28

**Authors:** Linan Ma, Luhu Ma, Xiaoguang Ma, Cunlong Zhou, Zhengyi Jiang, Jingwei Zhao

**Affiliations:** 1 School of Mechanical Engineering, Taiyuan University of Science and Technology, Taiyuan 030024, China; linanma@tyust.edu.cn (L.M.);; 2 College of Mechanical and Vehicle Engineering, Taiyuan University of Technology, Taiyuan 030024, China; 3 Engineering Research Center of Advanced Metal Composites Forming Technology and Equipment, Ministry of Education, Taiyuan 030024, China; 4 National Key Laboratory of Metal Forming Technology and Heavy Equipment, Ministry of Science and Technology, Taiyuan 030024, China; 5 School of Mechanical, Materials, Mechatronic and Biomedical Engineering, University of Wollongong, Wollongong, NSW 2522, Australia

**Keywords:** nanolubricants, titanium foils, micro rolling, tribological behavior, lubrication mechanisms

## Abstract

The tribological behavior of traditional oil-in-water (O/W) lubricants (1.0 wt.%) and nano-TiO_2_ additive lubricants (1.0–9.0 wt.%) during micro rolling of titanium foils were analyzed. In this study, the surface morphologies of titanium foils under various lubrication conditions were assessed, and the corresponding lubrication mechanisms were revealed. The tribological behavior of nano-TiO_2_ additive lubricants during micro rolling of titanium foils was also explored through a series of characterization methods. The utilization of nano-TiO_2_ additive lubricants in micro rolling reduces the surface roughness of titanium foils. Moreover, it effectively inhibits the generation of indentations and cracks during rolling processes, enhancing the surface quality of rolled specimens. Additionally, owing to the synergism of rolling, tribo-film, mending and polishing effects of the nanoparticles, both the rolling force and surface roughness were minimized by using lubricants containing 3.0 wt.% TiO_2_ nanoparticles. Overall, an optimal concentration (3.0 wt.%) of TiO_2_ nanoparticles in water-based nanolubricants was obtained with enhanced tribological properties and lubrication performance during micro rolling of titanium foils.

## 1. Introduction

Owing to superior biocompatibility and high corrosion resistance, titanium and its alloys are widely used to fabricate micro parts applied in aerospace engineering, medical science, and automotive industries [1]. Micro rolling is a key technique in the production of micro-components with high forming efficiency and environmental compatibility [2]. However, severe plastic deformation generated during micro rolling causes marked undulations on the titanium foils. Therefore, lubricants are applied to rolling processes to enhance the quality of titanium foils. Although traditional oil-based lubricants are known for their excellent tribological properties and lubrication performance, the environmental pollution induced by oil-based lubricants should never be ignored [3]. The exploration of the environmentally friendly nanolubricants with outstanding lubrication characteristics, therefore, is crucial for the development of manufacturing engineering.

Nano-lubricating material refers to a new type of lubricating material containing nano-state lubricating particles with the size between 1 and 100 nm, which has emerged as a critical area of research with significant implications for various industries, including automotive, aerospace, and manufacturing [4]. Graphene-based lubricants have demonstrated exceptional anti-wear and anti-friction properties, making them promising candidates for reducing mechanical wear and energy losses in automotive engines and industrial machinery [5]. Furthermore, the use of nanodiamond additives in lubricants has shown to enhance the durability and load-carrying capacity of lubricated surfaces, offering potential benefits for extending the service life of mechanical components [6]. Moreover, the application of nanolubricants in the aerospace sector has garnered attention for their ability to mitigate frictional losses and reduce fuel consumption in aircraft engines and propulsion systems [7]. Overall, the field of nanolubricants presents a promising avenue for improving the performance and longevity of mechanical systems across various industries, paving the way for future advancements in tribology and lubrication science.

The utilization of nanoparticles in lubricants has attracted considerable attention owing to their potential to enhance the wear resistance and efficiency of mechanical systems [8,9,10]. Kumar et al. [11] conducted a comprehensive review of nanolubricants and tribological behavior of the rolling process, highlighting the potential benefits of using nanoparticles in lubrication. Kamal et al. [12] investigated the preparation, characterization, and tribological behavior of lube oil with nanoparticle additives, further demonstrating the potential of nanotechnology in lubrication. Padgurskas et al. [13] studied the lubrication performance of mineral oil lubricants containing Fe, Cu, and Co nanoparticles. It was found that the lubricants containing nanoparticles effectively reduced the coefficient of friction (COF). Peng et al. [14] assessed the frictional characteristics of diamond and nano-SiO_2_. The results suggested that the wear resistance characteristics of the liquid paraffin with nanoparticles was superior compared to the pure paraffin oil. In addition, the tribological behavior of polyalphaolefin (PAO 6) with CuO, ZrO_2,_ and ZnO nanoparticles was reported by Battez et al. [15]. It was found that nanoparticle suspensions exhibited superior frictional characteristics compared to traditional base oil. Noori et al. [16] evaluated the frictional characteristics of nanolubricants by using a pin-disk setup. The results showed that adding CuO and MoS_2_ nanoparticles to castor oil and moulding oil exhibited a tribo-film effect which effectively enhances anti-wear properties. Xia et al. [17] proposed the modified oxide scale growth model to explore the influence of oxide scale surface morphology on the tribological properties of nano-TiO_2_ additive oil-in-water (O/W) lubricants by ball-on-disk tests. The results showed the tribo-sintering effect of TiO_2_ occurred when the TiO_2_ nanoparticles entered the friction pairs, repairing the worn scar of materials. Alghani et al. [18] studied the tribological behavior of graphene and TiO_2_ by a four-ball test. It was found that the blend of nanolubricants significantly improves wear scars. Ahmed et al. [19] compared the tribological performance of ZnO nanolubricants and 10W-40 engine oi. It was found that ZnO nanolubricants produced smoother rubbing surfaces. The tribological characterization of CeO_2_ nanoparticles was conducted by Singh et al. [20]. The results suggested that CeO_2_ nanoparticles demonstrated a reduction in COF. Additionally, Zenebe et al. [21] explored the anti-friction performance of ZrO_2_/MoDTC additives on a pin-on-disc tribotester. It was found that the composite nanolubricants could function with a synergistic lubrication effect during the friction process.

The above reports demonstrated that nano-additives can effectively enhance the wear resistance and frictional characteristics of lubricants. Consequently, the utilization of nanolubricants in rolling processes has been explored by many scholars. The effects of nano-SiO_2_ on the surface morphology, oxide scale, and microstructure of hot rolled steel strips were studied by Bao et al. [22,23], and it was found that the nano-SiO_2_ lubricants enhanced the surface quality of strips owing to the synergism of self-repairing and polishing functions of the nanoparticles. Sun et al. [24] evaluated the lubrication performance of MoO_3_ nanofluid applied in cold rolling of steel, and found that lubricating film on the contact area by electron transfer and deposition improves the surface quality of rolled strips. Sreeram et al. [25] investigated the properties of aluminum alloy composites reinforced with SiC nanoparticles prepared in a hot rolling process. It was found that the tensile strength of hot rolled composites is significantly increased. A novel MoS_2_-Al_2_O_3_ water-based nanofluid was prepared by He et al. [26,27]. It was found that the synergistic effect of MoS_2_ and oxidized MoO_3_ particles contributes to improved lubrication properties. Huo et al. [28] conducted micro flexible rolling experiments under different lubrication conditions, and few rolling marks were observed on the surface of aluminum specimens owing to the synergistic mechanisms including rolling, tribo-film, polishing, and mending effects. Roy et al. [29] studied the micropitting and wear behavior of CuO and WC nanofluids under boundary lubrication conditions by micropitting tests, and found that CuO performed slightly better on micropitting and wear resistance. Wang et al. [30,31] synthesized a reduced graphene oxide-Al_2_O_3_ nanolubricants and performed a series of cold rolling experiments. Nanoparticles were found to act as interlayer sliding, rolling, polishing, and mending between the rolls and specimens, which reduced COF during rolling processes.

In our previous work, the lubrication performance of nano-TiO_2_ additive lubricants during micro rolling of copper foils was studied, and it was found that a concentration of 3.0 wt.% nano-TiO_2_ additive lubricants contributes to the improved lubrication performance [32]. In view of the excellent lubrication performance of the prepared nanolubricants, it is necessary to study the rolling effect of nanolubricants applied to different materials and compare the lubrication performance with traditional O/W lubricants. Although the lubricating effect of nanolubricants has been studied by many scholars, the application of nanolubricants during rolling of pure titanium foils has been rarely involved. In this study, the traditional O/W lubricants and nano-TiO_2_ additive lubricants were prepared and the tribological behavior of nano-TiO_2_ additive lubricants during micro rolling of titanium foils was explored. This work aims to clarify the effect of nano-TiO_2_ additive lubricants in micro rolling of titanium foils and illustrate the lubrication mechanisms of TiO_2_ nanoparticles during micro rolling processes.

## 2. Experimental Procedures

### 2.1. As-Received Material

Pure titanium foils with the dimensions of 150 × 5 × 0.1 mm^3^ were used for micro rolling tests in the present work. Table 1 shows the chemical compositions of the as-received foils. Before the experiment, the specimens were wiped with alcohol to eliminate the effect of the original surface conditions.

### 2.2. Preparation of Nanolubricants

**Figure 1:**
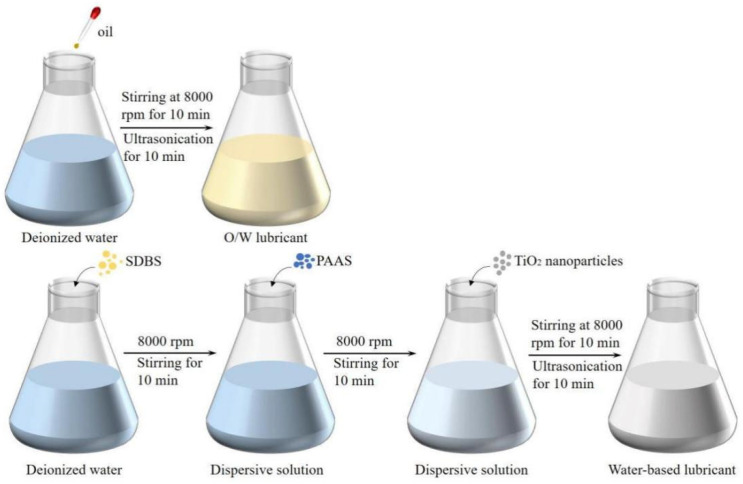
Flow chart of preparing O/W lubricants and nano-TiO_2_ additive lubricants.

In this work, nanolubricants were prepared with TiO_2_ nanoparticles (purity > 99.9%, 30 nm) as additives. Figure 1 shows the preparation process of the O/W lubricants and nano-TiO_2_ additive lubricants. For O/W lubricants, 1.0% oil was added to deionized water and dispersed for 10 min, followed by ultra-sonication for 10 min. For the nano-TiO_2_ additive lubricants, first, sodium dodecylbenzene sulfonate (SDBS) was added to deionized water and dispersed at 8000 rpm for 10 min. SDBS is an effective anionic surfactant with molecules consisting of hydrophilic ionic heads and hydrophobic carbon chains that provide an electrostatic effect to prevent nanoparticle agglomeration, which is used as a dispersant in the formulation of this lubricants. Then, polyacrylic acid sodium salt (PAAS) and TiO_2_ nanoparticles were added sequentially to a dispersive solution and dispersed at 8000 rpm for 10 min. Finally, the obtained solution was dispersed with an ultrasonic disperser to ensure uniform dispersion of the remaining polymer. The prepared nano-TiO_2_ additive lubricants exhibited outstanding stability and no precipitation was observed during 5 days of aging.

The various lubrication conditions applied to micro rolling experiments are shown in Table 2, and the lubrication effects of different lubricants were thus obtained. The nano-TiO_2_ additive lubricants with different concentrations (1.0~9.0 wt.%) and SDBS with corresponding concentration were used. The nano-TiO_2_ additive lubricants (3.0 wt.%) reported the best values for the performance parameters considered [33]. The concentrations of nano-TiO_2_ additives based on titanium foils were selected at 1%, 3%, 5%, 7%, and 9% in order to provide a comprehensive comparative analysis with prior research that utilized similar concentrations of nano-TiO_2_ additives. By including these specific values, direct comparisons were made and meaningful conclusions were drawn regarding the performance and characterization of the nano-TiO_2_ additives in the context of this study. This approach allowed for a more thorough assessment of the impact of varying concentrations of nano-TiO_2_ additives on the properties under investigation. Nano-TiO_2_ additive lubricants do not contain oil or other toxic substances, which prevents environmental pollution and damage to the substrate.

### 2.3. Micro Rolling Tests

**Figure 2:**
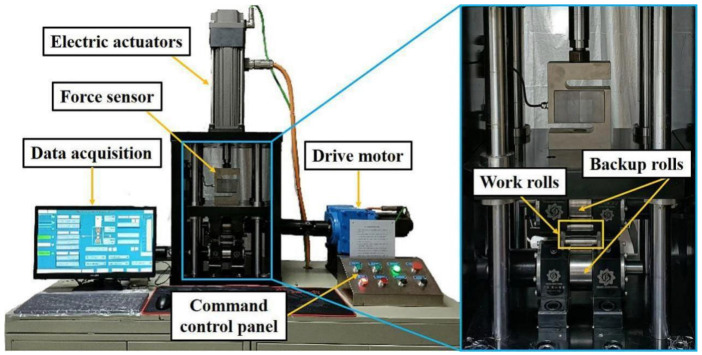
Rolling mill used in the present work.

**Figure 3:**
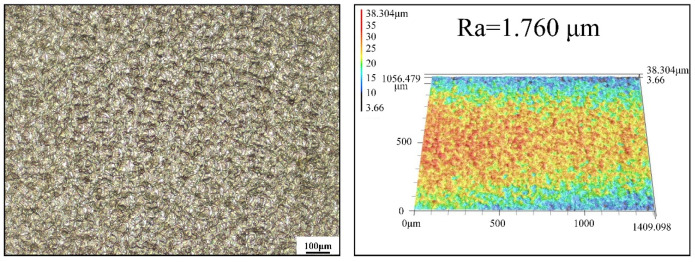
Surface morphology and 3D profile of the work roll.

Figure 2 shows the rolling mill used to carry out the micro rolling experiments, and the surface morphology and three-dimensional profile of the working roll used in this work are shown in Figure 3. A series of rolling experiments were conducted with the rolling speed of 1 m/min and the reduction of 10%. The lubricants were uniformly applied to the rolls by spraying before each experiment. Three specimens were tested under each lubrication condition to obtain an average value, which eliminated errors generated during experiments. Several small samples with 6 mm in length were cut along the rolling direction (RD) from the rolled specimens for subsequent observation and measurement.

### 2.4. Characterization and Analytical Approaches

The 2D surface morphologies and 3D topographies of the rolled titanium foils were detected by a KEYENCE VK-X1000 3D laser scanning microscope (Keyence Corporation, Okasa, Japan). The surface roughness of six randomly selected locations along the RD were measured and analyzed. A JEOL-IT500 scanning electron microscope (SEM) (JEOL, Tokyo, Japan) equipped with an energy dispersive spectroscopy (EDS) detector (Oxford Ltd., Oxford, UK) was used to obtain the elemental distribution on the surface of rolled titanium foils. The nanoparticle distribution and the microscopic morphology of the titanium foils were observed by SEM as well.

## 3. Results and Discussion

### 3.1. Rolling Force and Surface Roughness

**Figure 4:**
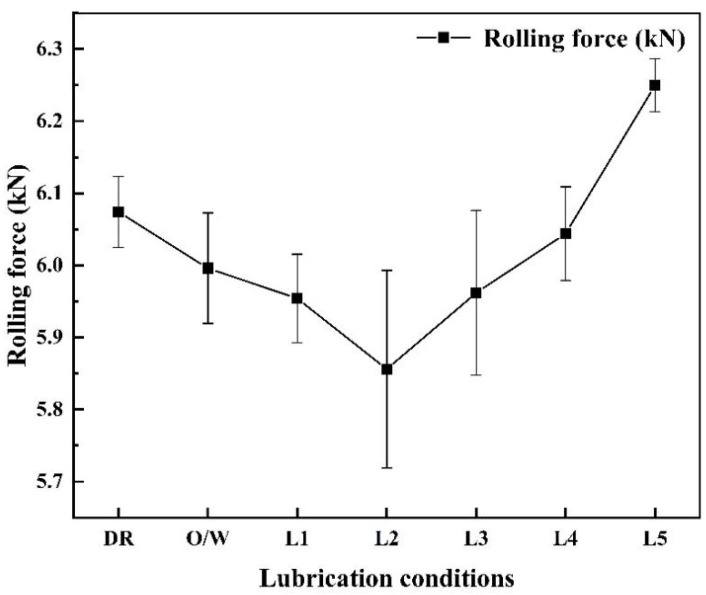
The rolling forces of titanium foils rolled with various lubrication conditions.

Figure 4 shows the rolling forces during the micro rolling of titanium foils with various lubrication conditions. Compared with the DR condition, O/W lubricants and nano-TiO_2_ additive lubricants can effectively reduce the rolling force. For nanolubricants, the rolling force decreased from 5.954 to 5.856 kN when the concentration of TiO_2_ nanoparticles increased from 1.0 to 3.0 wt.%, which can be attributed to the reduced energy consumption induced by nanoparticles [28]. Nevertheless, the rolling force increased from 5.856 to 6.250 kN with the further increasing concentration of TiO_2_ nanoparticles from 3.0 to 9.0 wt.%. This phenomenon can be explained by the aggregated nanoparticles [34]. In general, excessive nanoparticles can lead to increased friction, thereby increasing the COF and leading to an increase in rolling force [35].

**Figure 5:**
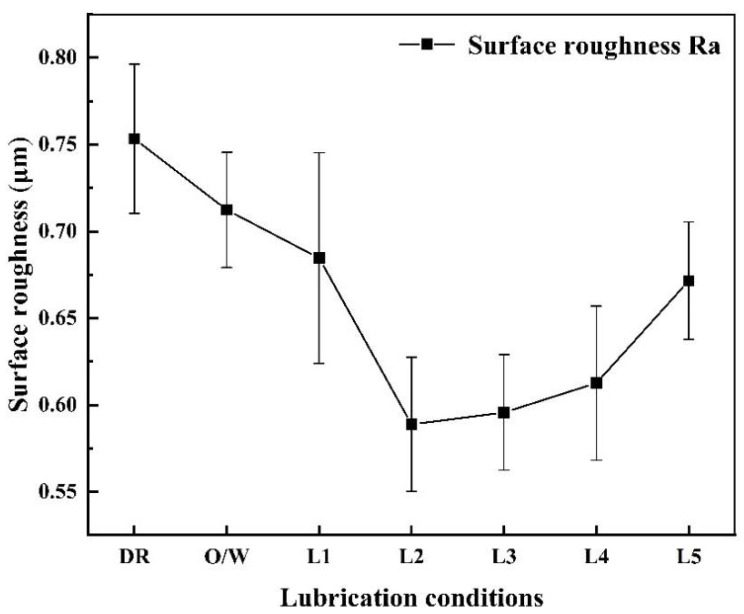
Surface roughness of titanium foils rolled with various lubrication conditions.

The surface roughness of titanium foils after rolling with various lubrication conditions is shown in Figure 5. Titanium foils exhibited the highest surface roughness under the DR condition. In addition, it was seen that nano-TiO_2_ additive lubricants improved the surface roughness more remarkably than under the O/W lubrication condition. When using 3.0 wt.% nano-TiO_2_ additive lubricants, the lowest Ra value was achieved, indicating the superiority of nano-TiO_2_ additive lubricants. Nevertheless, the surface roughness of the titanium foils increased gradually when further increasing the concentration of TiO_2_ nanoparticles from 3.0 wt.% to 9.0 wt.%. The phenomenon can be explained by the smaller contact area due to the aggregation of nanoparticles [36].

### 3.2. Surface Profile and 3D Morphologies

**Figure 6:**
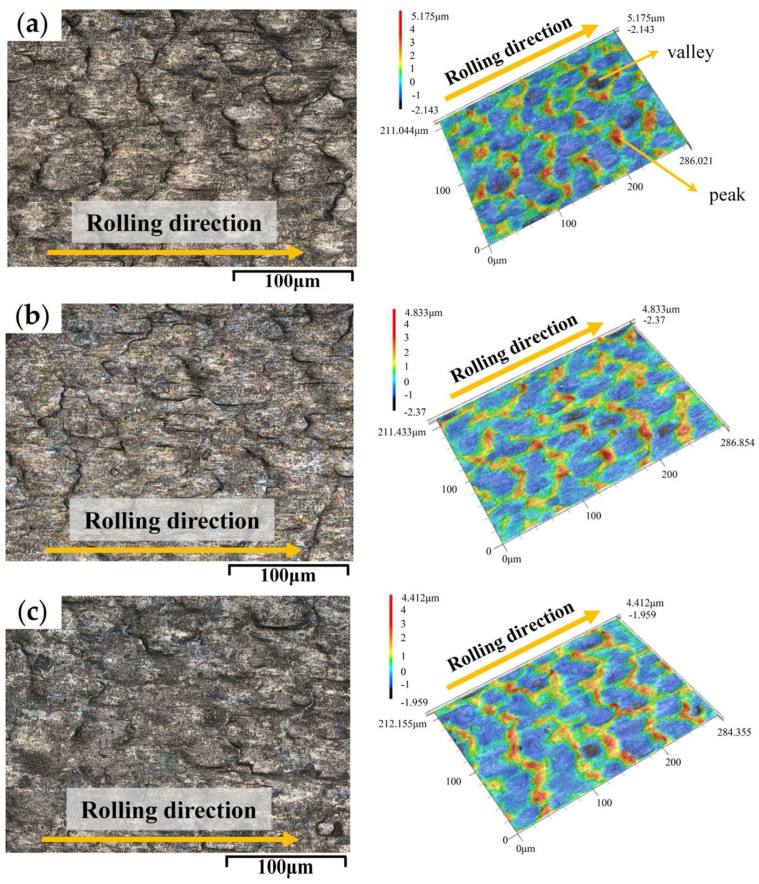

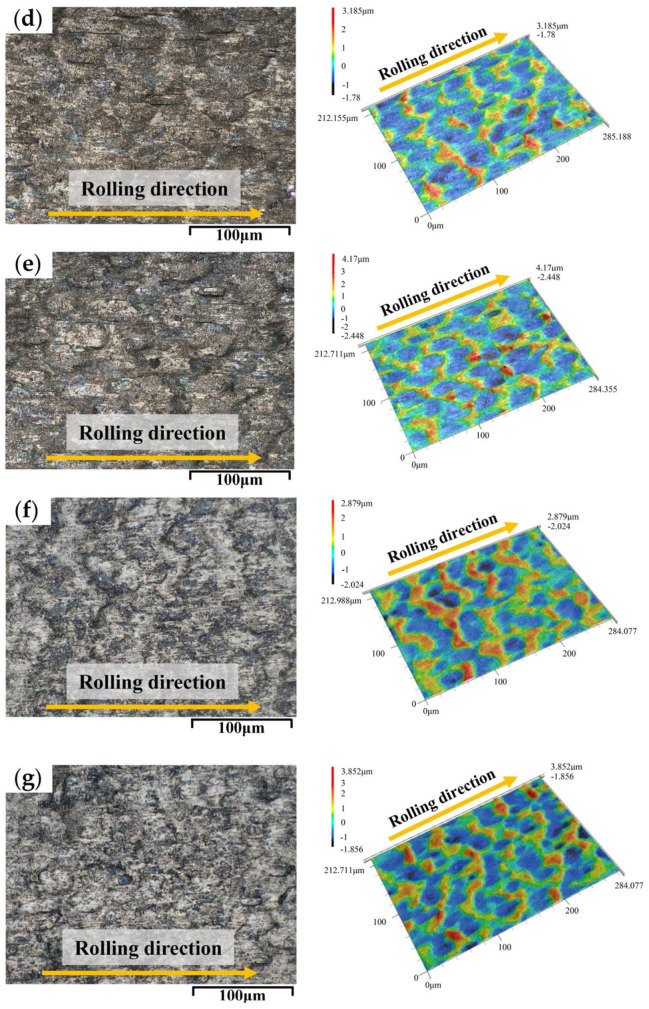
Surface profile and 3D morphologies of the rolled titanium foils with various lubrication conditions: (**a**) DR, (**b**) O/W, (**c**) L1, (**d**) L2, (**e**) L3, (**f**) L4, (**g**) L5.

**Figure 7:**
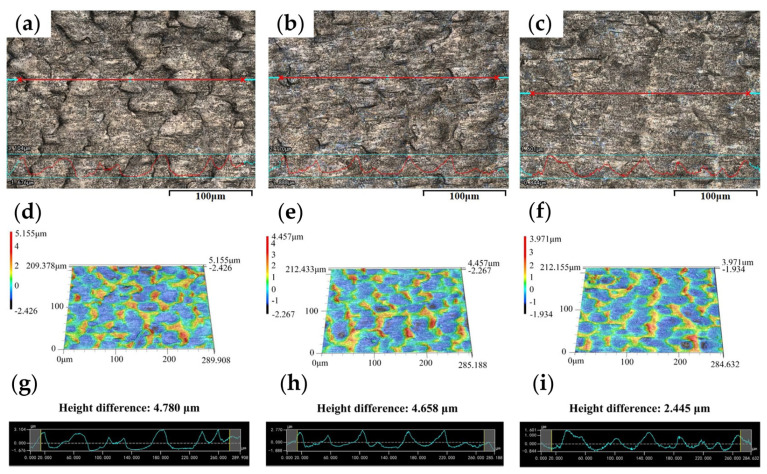
The surface profile, 3D morphologies, and corresponding height morphologies of rolled strips with various lubrication conditions: (**a**,**d**,**g**) DR, (**b**,**e**,**h**) O/W, (**c**,**f**,**i**) L2.

Figure 6 shows the detailed surface morphologies of the rolled titanium foils. Under the dry condition, the surface of specimen undergoes severe plastic deformation during micro rolling, promoting the formation of peaks and valleys on the specimens. The improved surface quality was obtained with the concentration of 3.0 wt.% TiO_2_ nanoparticles, indicating that the application of nano-TiO_2_ additive lubricants is beneficial to the enhancement of the surface quality of rolled titanium foils. Figure 6g shows the surface morphology of rolled strips with the concentration of TiO_2_ nanoparticles ranging from 3.0 wt.% to 9.0 wt.%. It was found that the surface of the specimen deteriorated severely as more undulations appeared, which matches well with the results shown in Figure 5. The surface profile, 3D morphologies, and corresponding height morphologies of the rolled titanium foils under DR, O/W, and L2 conditions are shown in Figure 7. It can be seen that the surfaces rolled with 3.0 wt.% nanolubricants exhibited notably smoother condition with the lowest height difference, indicating the enhanced lubrication effect induced by TiO_2_ nanoparticles.

### 3.3. SEM-EDS Analysis

**Figure 8:**
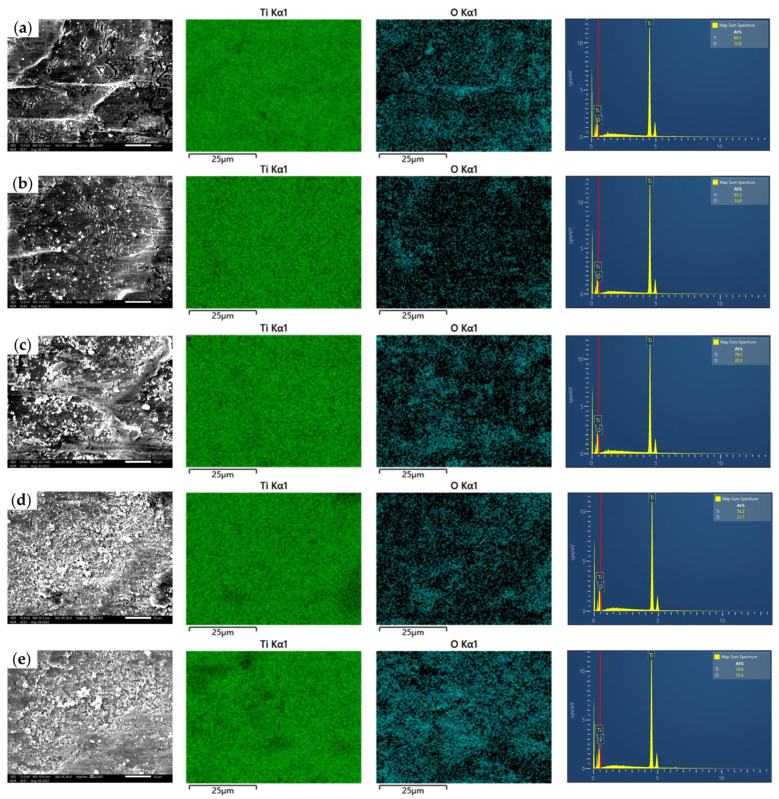
The SEM and EDS mappings of the rolled titanium foils with various concentrations of nano-TiO_2_ additive lubricants: (**a**) 1.0 wt.%, (**b**) 3.0 wt.%, (**c**) 5.0 wt.%, (**d**) 7.0 wt.%, (**e**) 9.0 wt.%.

Figure 8 shows the SEM and EDS mappings of the rolled titanium foils lubricated with nanolubricants containing different concentration of TiO_2_ nanoparticles. The distribution of nanoparticles can be roughly observed through the distribution of O elements in the EDS images. For specimens rolled with lubricants containing 1.0 wt.% of TiO_2_ nanoparticles (as shown in Figure 8a), few nanoparticles could be observed on the surface of foils. For specimens rolled with lubricants containing 3.0 wt.% TiO_2_ nanoparticles (as shown in Figure 8b), more nanoparticles were distributed on the surface of the rolled foils, which effectively improved lubrication effects. Nevertheless, for specimens rolled with lubricants containing TiO_2_ nanoparticles ranging from 5.0 to 9.0 wt.% (as shown in Figure 8c–e), aggregated nanoparticles appeared and deteriorated the surface of titanium foils. Therefore, 3.0 wt.% nano-TiO_2_ additive lubricants exhibit the best lubrication effect, and are therefore optimal for micro rolling of titanium foils.

**Figure 9:**
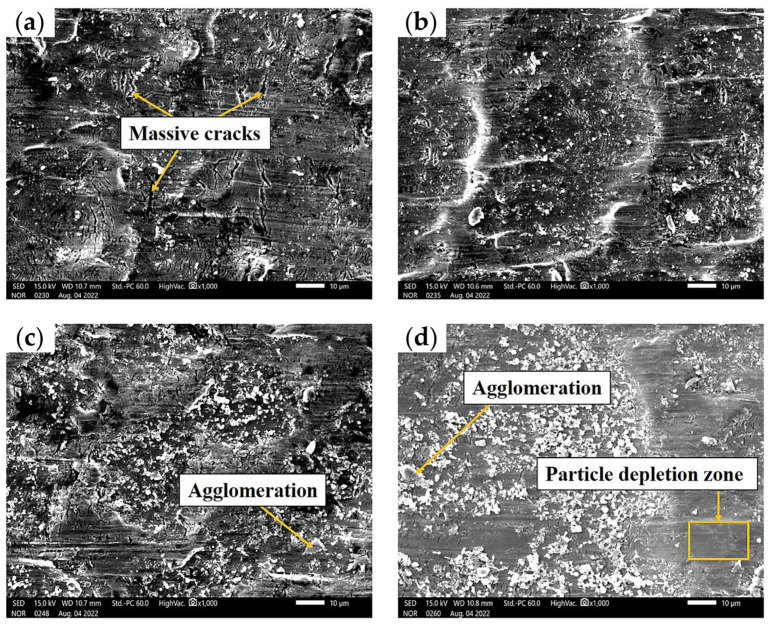
The SEM images of rolled titanium foils under various concentrations of nano-TiO_2_ additive lubricants: (**a**) 1.0 wt.%, (**b**) 3.0 wt.%, (**c**) 5.0 wt.%, (**d**) 9.0 wt.%.

To explore the macroscopic distribution of TiO_2_ nanoparticles on the rolled surface, the SEM images with lower magnification (×1000) of the rolled titanium foils lubricated by nanolubricants containing different concentration of TiO_2_ nanoparticles were obtained, as shown in Figure 9. The deep groove cracks and nanoparticle distributions can be clearly seen from the SEM images. When lubricated under the L1 condition, massive cracks were clearly observed on specimens. When the concentration of TiO_2_ nanoparticles increased to 3.0 wt.% (L2 condition), the nanoparticles were uniformly dispersed on the contact area without obvious aggregation, and the best surface morphology was achieved on rolled foils. However, obvious aggregation of nanoparticles could be observed when the concentration of TiO_2_ nanoparticles increased to 9.0 wt.% (L5 condition). It is noteworthy that an excessive amount of nanoparticles in lubricant leads to the formation of particle depletion zones on the surface morphologies. The main reason is that the aggregated nanoparticles support the pressure during micro rolling, while the high concentration of nanoparticles cannot stay or embed in the rolling surface after a light rubbing with alcohol [28].

### 3.4. Lubrication Mechanisms

**Figure 10:**
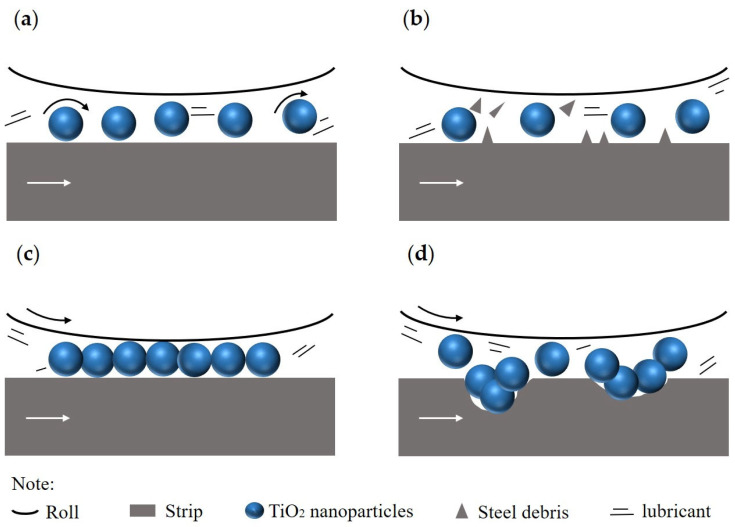
The schematic diagram of the lubrication mechanisms of nano-TiO_2_ additive lubricants during micro rolling process: (**a**) rolling effect, (**b**) polishing effect, (**c**) tribo-film effect, (**d**) mending effect.

The lubrication mechanisms of nanoparticles in the water-based nanolubricants have been systematically explored during past decades. At present, four lubrication mechanisms, that is, the rolling effect [37], the polishing effect [38], the tribo-film effect [39], and the mending effect [40], have been widely recognized. Figure 10 shows the diagram of the mentioned mechanisms. During rolling of titanium foils, the rolling effect enhances the load bearing capacity by transitioning from the sliding friction to the rolling friction at the contact interface, thereby effectively reducing the COF, as shown in Figure 10a. Additionally, TiO_2_ nanoparticles with high hardness are supposed to effectively grind the surface bumps of the foils during the rolling process. This grinding action functions to reduce the surface roughness and enhance the forming quality of the rolled foils, as shown in Figure 10b. For the tribo-film effect, a thin but dense tribo-film is formed between the specimens and the work roll, which isolates the roll from direct contact with the processed material, thus improving the wear resistance of specimens, as shown in Figure 10c. In addition, the nanoparticles enter into microdefects or deep grooves on the surface of foils, and this mending effect promotes the formation of smooth surface during rolling, as shown in Figure 10d.

**Figure 11:**
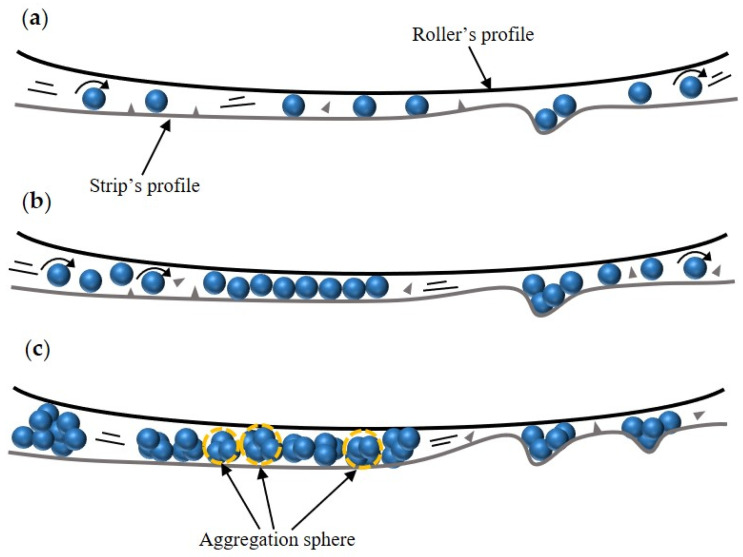
Speculative lubrication mechanisms with different concentrations of nanoparticles acting as additives at the roll-strip contact interface: (**a**) 1.0 wt.%, (**b**) 3.0 wt.%, (**c**) 9.0 wt.%.

Figure 11 shows the speculative lubrication mechanisms in micro rolling processes when using nano-TiO_2_ additive lubricants. For the titanium foils rolled with lubricants containing 1.0 wt.% TiO_2_ nanoparticles (as shown in Figure 11a), few nanoparticles entered the contact area and led to insufficient lubrication effects. Noticeably, more nanoparticles entered the contact area when lubricants containing 3.0 wt.% TiO_2_ nanoparticles (as shown in Figure 11b), forming a thin and dense tribo-film with low shear, which effectively prevented the direct contact between friction pairs and improved the lubrication effects. The high hardness nanoparticles can polish the bumps and deposit in the grooves of the worn surface, thereby improving the surface quality. For the titanium foils rolled with lubricants containing 9.0 wt.% TiO_2_ nanoparticles (as shown in Figure 11c), the nanoparticles tended to aggregate at the roll-strip contact interface, and the severe agglomeration suppressed the continuous supply of the lubricant to the friction area, causing a great loss of lubricant [41]. After applying nano-TiO_2_ additive lubricants between the titanium foils and the work roll, nanoparticles tend to stay at contact area and isolate the roll from direct contact with titanium foils. Moreover, the excessive amount of nanoparticles affects the stability of the tribo-film, causing a deterioration in the surface of rolled specimens [42]. The unstable lubricating film hardly remains on the surface of the titanium foil, thereby leading to the formation of particle depletion zones. In addition, a dense tribo-film and spherical nanoparticles isolate titanium foils and the work roll, resulting in a reduced COF. It has been reported that lubricants with higher composition contribute to improved lubrication effects (such as enhanced rolling effect and tribo-film effect), preventing direct contact between friction pairs during or after deformation [28]. Additionally, nanoparticles exhibit a repairing effect on the surface of titanium foil by depositing into deep grooves and cracks during micro rolling. Therefore, optimization in the concentration of TiO_2_ nanoparticles of water-based lubricants is crucial for improving lubrication conditions during rolling processes. Overall, 3.0 wt.% nano-TiO_2_ additive lubricants exhibit improved lubrication performance, enhancing the surface quality of rolled titanium foils during micro rolling.

## 4. Conclusions

### Summary

In this manuscript, the tribological behavior of nano-TiO_2_ additive lubricants during micro rolling of titanium foils was analyzed, and the lubrication mechanisms were revealed. The following conclusions can be drawn:

1. A nano-TiO_2_ additive lubricant was developed and applied to micro rolling to enhance the surface quality of rolled titanium foils. Nano-TiO_2_ additive lubricants exhibited improved lubrication performance during micro rolling compared to the traditional O/W lubricants, confirming the superiority of nanolubricants compared to traditional O/W lubricants during lubrication.
2. An optimal concentration of 3.0 wt.% nano-TiO_2_ additive lubricants was found for water-based nanolubricants, which agrees well with expectation. The application of prepared lubricant contributed to a remarkable reduction in the surface roughness of rolled titanium foils (~21.8%), which is mainly attributed to the synergism of the polishing and the mending effects of the nanoparticles.
3. During micro rolling, the dense tribo-film and spherical nanoparticles isolates the working roll from direct contact with the titanium foils, which confirms the lubrication mechanism of nanolubricants. As a result, the TiO_2_ nanoparticles at the roll-strip contact interface inhibit the generation of cracks of titanium foils, enhancing the surface quality of rolled products.

## Figures and Tables

**Table 1:** Chemical compositions of the as-received titanium foils (wt.%).

Element	Ti	Fe	N	H	O	C
Content	≥99.6	≤0.014	≤0.008	≤0.0013	≤0.046	≤0.010

**Table 2:** Lubrication conditions applied to micro rolling tests.

Lubricant Conditions	Description
DR	Dry
O/W	1.0 wt.% oil + balance water
L1	1.0 wt.% TiO_2_ + 0.2 wt.% SDBS + 0.3 wt.% PAAS + balance water
L2	3.0 wt.% TiO_2_ + 0.6 wt.% SDBS + 0.3 wt.% PAAS + balance water
L3	5.0 wt.% TiO_2_ + 1.0 wt.% SDBS + 0.3 wt.% PAAS + balance water
L4	7.0 wt.% TiO_2_ + 1.4 wt.% SDBS + 0.3 wt.% PAAS + balance water
L5	9.0 wt.% TiO_2_ + 1.8 wt.% SDBS + 0.3 wt.% PAAS + balance water

## Data Availability

Data are contained within the article.
